# Early and mid-term outcomes of the aortic arch surgery: experience from the low-volume centre

**DOI:** 10.1186/s13019-015-0229-6

**Published:** 2015-03-10

**Authors:** Radim Brat, Jaroslav Gaj, Jiri Barta

**Affiliations:** 1 Department of Cardiac Surgery, University Hospital in Ostrava, 17. listopadu 1790, 708 52 Ostrava, Czech Republic; 2 Faculty of Medicine, University of Ostrava, Ostrava, Czech Republic

**Keywords:** Aortic arch surgery, Low-volume centre, Caseload, Cerebral protection

## Abstract

**Aim:**

The aim of this retrospective study was to examine the early and mid-term outcomes for patients undergoing elective aortic arch surgery over a 13-years period in the single low-volume centre. Results of aortic arch surgery published in the literature are usually results of high-volume centers, but the majority of institutions have much lower caseload.

**Methods:**

From January 1999 to March 2013 total of 353 surgeries on thoracic aorta were performed in our institution. Only 30 procedures (8.5%) were elective aortic arch surgeries. This group of patients was analyzed.

**Results:**

Deep hypothermia alone and hypothermia with ortograde cerebral perfusion was used in 7 (23%) and 23 (77%) patients respectively. Mean core temperature was 22°C (17 – 26°C). Cannulation sites was axillary artery or brachiocephalic trunk in 17 (57%), femoral artery in 8 (27%) and ascending aorta or aortic arch in 5 (16%). Mean hypothermic circulatory arrest time was 39 min (15 – 74 min). There was one death due to multiorgan failure; all-cause mortality at 30 days was 3.3%. The frequency of other complications was permanent neurological deficit in 2 (6.7%), temporary neurological deficit in 2 (6.7%) and renal failure requiring hemodialysis in 2 (6.7%) patients. In the follow-up 13 patients died, remaining 16 are still alive.

**Conclusion:**

Despite the lower caseload and technical problems manifested by a higher number of re-operations for bleeding, the all-cause mortality at 30 days as well as mid-term results are comparable with results reported by the high-volume centres.

## Background

There has been great interest in general and cardiac surgery with respect to the relationship between caseload and clinical outcome [1]. It has been suggested that mortality rates might be reduced by 20–50% for a range of procedures including carotid endarterectomy, abdominal aortic aneurysm repair, pancreatectomy and oesophagectomy if carried out by high volume surgical groups [2]. It has been suggested that volume-based referral strategies are most appropriate for operative interventions, which are relatively infrequent, technically complex and with challenging post-operative care [1]. Surgery of the aortic arch would seem to be one area where such considerations might apply because surgical treatment of aortic arch pathology requires partial or complete replacement of the aortic arch while the systemic circulation is temporarily interrupted. Patients undergoing this obligatory period of circulatory arrest are at an increased risk for adverse neurologic outcomes and ischemic end-organ damage. Therefore, strategies for cerebral protection and circulation management must be implemented to achieve optimal clinical results [3].

Results of aortic arch surgery published in the literature are usually results of high-volume centers, performing more than 50 aortic arch surgeries per year [4-7]. But for practical reasons and because of limited mobility of patients the majority of institutions have much lower caseload in this specialized field of cardiac surgery. When comparing results of aortic arch surgery, we have to take into consideration the percentage of acute dissection in the series. Acute aortic dissection has a high early mortality with an exponential decline in survival within a short time. Transfer to a distant specialized centre may compromise overall survival. That is why we can expect higher percentage of dissections in low-volume centers negatively influencing their overall results.

This retrospective analysis examined early and mid-term outcomes of the elective aortic arch surgery in the low-volume centre. We believe these data could make a picture of results in the field of aortic arch surgery more complex.

## Methods

### Patient population

From January 1999 to March 2013 total of 353 surgeries on thoracic aorta were performed in our institution. Aortic pathology was aortic aneurysm in 212 (60.0%) cases, acute aortic dissection in 114 (32.3%) cases, chronic aortic dissection in 22 (6.2%) and other pathologies (infection etc.) in 5 (1.5%) cases. Circulatory arrest with cerebral protection was used in 140 (39.7%) cases. These numbers represent our overall caseload in this field of thoracic aorta surgery.

The vast majority of these patients (323 patients, 91.5%) were patients operated in the regime of acute operations and patients with aortic dissection. For the reasons mentioned in the introduction we excluded these patients from our study.

Only 30 procedures (8.5%) were elective aortic arch surgeries in patients with aortic arch aneurysm. This highly selected and homogeneous group of patients was included in our analysis and all data in this article are related to this group of patients.

### Data collection and definitions

Demographic, perioperative and follow-up data were collected prospectively on a computer database (National Adult Cardiac Database) and case-note review, both analyzed retrospectively. The follow-up was 100%.

Only those operations involving two or more distal anastomoses, one to the distal aorta and one or more aortic arch branches, were considered as aortic arch operations. In accordance with STS guidelines, early mortality was defined as all-cause mortality at 30 days. Neurological complications were defined as permanent neurological deficit (PND) for patients with stroke or paraplegia, and temporary neurological deficit (TND) for patients with reversible deficits.

### Operative techniques

Although we tried to have broad consensus with respect to the operative strategy, perfusion techniques and neuroprotective strategies, a range of surgical techniques was practiced over the 14-year period.

### Operative approach

All the operations were performed through a median sternotomy incision. Where improved access was required for extensive reconstructions of the distal aortic arch and/or the proximal descending aorta, a lateral T-extension into the left fourth intercostal space was used.

### Perfusion techniques

Cardiopulmonary bypass was established through cannulation of the femoral artery between 1999 and 2002. Since 2003 cannulation of axillary artery or brachiocephalic trunk was used. Venous drainage generally was provided by a two-stage cannula, only in patients with concomitant mitral or tricuspid valve surgery bicaval cannulation was used. Venting of the pulmonary artery was used in the majority of cases. Antegrade cold crystalloid or blood cardioplegia was used for myocardial protection. Between 1999 and 2000 deep hypothermic circulatory arrest with target cooling temperature 18–20°C was used. Since 2001, selective antegrade cerebral perfusion was used after the technique of Kazui et al. [8].

## Results

### Baseline characteristics

Baseline characteristics are summarized in Table 1. The median age was 60 years (range 31–80 years) with a male preponderance (57%). There were 4 (13%) patients with a history of previous cardiac surgery. Mean ejection fraction was 55% (range 35% - 70%). Chronic kidney disease with serum creatinine over 180 mmol/l was in 2 (6.7%) patients.

**Table 1:** **Baseline characteristics**

No. of patients	30
Age (years)	60 (31 – 80)
Male/Female	17/13
Reoperation	4 (13%)
EF (%)	55 (35 – 70)
Creatinin > 180 mmol/l	2 (6.7%)

EF….ejection fraction.

### Operative details

Details of the surgical procedure and operative details are summarized in Table 2.

**Table 2:** **Operative details**

Cross-clamp time (min)	103 (45 – 231)
Circulatory arrest (min)	39 (15 – 74)
Cerebral protection: DHCA	7 (23%)
DHCA + OCP	23 (77%)
Core temperature during arrest (°C)	22 (17–26)
Cannulation site: axillary artery or brachiocephalic trunk	17 (57%)
Femoral artery	8 (27%)
Ascending aorta or arch	5 (16%)

DHCA…deep hypothermia circulatory arrest.

OCP…ortograde cerebral perfusion.

Mean cross-clamp time was 103 min (range 45 – 231 min); mean hypothermic circulatory arrest time was 39 min (range 15 – 74 min). As a cerebral protection deep hypothermia alone was used in 7 (23%) patients and hypothermia with ortograde cerebral perfusion in remaining 23 (77%) patients. Mean core temperature during circulatory arrest was 22°C (range 17 – 26°C). The reason for such a wide range of body temperature during circulatory arrest is the fact that in patients operated in deep hypothermia alone the core temperature 17 – 22°C was used, but in patients operated with the use of ortograde cerebral perfusion the core temperature 23–26°C was used. Cannulation sites was axillary artery or brachiocephalic trunk in 17 (57%), femoral artery in 8 (27%) and ascending aorta or aortic arch in 5 (16%) patients. Concomitant procedures are summarized in Table 3. The most common concomitant procedure was aortic root replacement (Bentall or valve sparing) in 17 (57%) followed by CABG in 7 (23%) and mitral valve plasty in 2 (7%) patients. Mitral valve replacement, tricuspid valve plasty and carotid endarterectomy were performed as a concomitant procedure in 1 (3%) patient.

**Table 3:** **Concomitant procedures**

Aortic root replacement (Bentall or valve sparing)	17 (57%)
CABG	7 (23%)
MVP	2 (7%)
MVR	1 (3%)
TVP	1 (3%)
Carotid endarterectomy	1 (3%)

CABG…coronary artery bypass grafting.

MVP…mitral valve plasty.

MVR…mitral valve replacement.

TVP…tricuspid valve plasty.

### In-hospital outcomes

There was one death due to multiorgan failure; all-cause mortality at 30 days was 3.3%. Two patients (6.7%) suffered from permanent neurological deficit and another two (6.7%) from temporary neurological deficit. Renal failure requiring hemodialysis experienced 2 patients (6.7%). There was quite high percentage of re-operation for bleeding (11 re-operations in 8 patients), majority of them during the initial experience. An average postoperative blood loss was 1270 ml (200–5200 ml). Postoperative blood loss was significantly higher during the first 5-year period. An average postoperative blood loss in the period 1999–2004 and 2004–2013 was 1546 ml and 955 ml respectively. An average intubation time and post-operative hospital stay was 116 hours and 17 days respectively. All in-hospital outcomes are summarized in the Table 4.

**Table 4:** **In-hospital outcomes**

30-day mortality	1 (3.3%)
Permanent neurological deficit	2 (6.7%)
Temporary neurological deficit	2 (6.7%)
Hemodialysis	2 (6.7%)
Reoperation for bleeding	8 (27%)
Postoperative blood loss (ml)	1270 (200 – 5200)
Intubation (hours)	116 (5 – 912)
Postoperative hospital stay (days)	17 (7 – 68)

### Mid-term outcomes

Our mid-term outcomes are based on the 100% follow-up. The median follow-up of survivors was 6.74 years. Out of the 29 patients 13 died during the follow-up, remaining 16 are still alive. We do not know exact cause of death in all of 13 patients who died during the follow-up period, but we know, that none of them died because of thoracic pathology. The average survivor time of patients who died during follow-up period was 5.25 (0.2 – 13.3) years. Three patients experienced re-intervention during the follow-up; it was descending aorta stentgrafting in all of them.

## Discussion

This study examined the early and mid-term outcomes for patients undergoing elective aortic arch surgery over a 13-years period in the single low-volume centre. The reason for that was the fact, that results of aortic arch surgery published in the literature are usually results of high-volume centers, performing more than 50 aortic arch surgeries per year. But for practical reasons and because of limited mobility of patients the majority of institutions have much lower caseload in this specialized field of cardiac surgery. When comparing results of aortic arch surgery, we have to take into consideration the percentage of acute dissection in the series. Acute aortic dissection has a high early mortality with an exponential decline in survival within a short time. Transfer to a distant specialized centre may compromise overall survival. That is why we can expect higher percentage of dissections in low-volume centers negatively influencing their overall results. We included only elective cases into our study. The negative impact of that is that the total number of patients included in the study is quite limited; on the other hand it is quite homogenous group of patients.

There was one perioperative death in our group of patients; all-cause mortality at 30 days was 3.3%. These numbers are comparable with data published in the literature [9] showing that also low-volume centre can achieve acceptable results in terms of all-cause mortality. Percentage of patients with permanent or temporary neurological deficit was slightly higher, than reported in the recent literature. But the majority of these patients were operated during our initial experience using deep hypothermic circulatory arrest with target cooling temperature 18–20°C as a brain protection method. Use of hypothermia with ortograde cerebral perfusion has improved the neurological results significantly. The average postoperative blood loss in our patients was high, especially during the initial experience. The reason for that was a lack of experience in the beginning of the series. There was a limited number of patients with extreme blood loss in the postoperative period making the average blood loss quite high. The same reason is for a very high percentage of patients requiring re-operation for bleeding. This is a sign of lack of experience in the beginning of the series as well. On the other hand these technical problems were solved successfully and didn’t influence the overall mortality and morbidity.

Our mid-term outcomes are based on the 100% follow-up. The mid-term outcomes are similar to those reported in the literature. Mid-term survival rate is acceptable and is influenced first of all by the concomitant diseases. None of our patients experienced surgical re-intervention in the mid-term folow-up, showing the surgical treatment was radical and durable enough, which is not surprising. Three patients required descending aorta stentgrafting as a second stage procedure.

## Conclusions

The aim of this retrospective study was to examine the early and mid-term outcomes for patients undergoing elective aortic arch surgery over a 13-years period in the single low-volume centre. The result of this study is, that despite the lower caseload and technical problems manifested by a higher number of re-operations for bleeding, the all-cause mortality at 30 days as well as mid-term results are comparable with results reported by the high- volume centers. We conclude that the caseload is definitely important for the experience of individual surgeon as well as the institution but on the other hand also low-volume centre can achieve acceptable results in the field of aortic arch surgery.

## Abbreviations

CABG: Coronary artery bypass grafting
DHCA: Deep hypothermia circulatory arrest
EF: Ejection fraction
MVP: Mitral valve plasty
MVR: Mitral valve replacement
OCP: Ortograde cerebral perfusion
PND: Permanent neurological deficit
STS: The Society of Thoracic Surgeons
TND: Temporary neurological deficit
TVP: Tricuspid valve plasty
